# A Sero-epidemiological Approach to Explore Transmission of *Mycobacterium ulcerans*

**DOI:** 10.1371/journal.pntd.0004387

**Published:** 2016-01-25

**Authors:** Kobina Assan Ampah, Beatrice Nickel, Prince Asare, Amanda Ross, Daniel De-Graft, Sarah Kerber, Ralf Spallek, Mahavir Singh, Gerd Pluschke, Dorothy Yeboah-Manu, Katharina Röltgen

**Affiliations:** 1 Swiss Tropical and Public Health Institute, Molecular Immunology, Basel, Switzerland; 2 University of Basel, Basel, Switzerland; 3 Noguchi Memorial Institute for Medical Research, University of Ghana, Legon, Ghana; 4 LIONEX Diagnostics & Therapeutics, Braunschweig, Germany; University of Tennessee, UNITED STATES

## Abstract

The debilitating skin disease Buruli ulcer (BU) is caused by infection with *Mycobacterium ulcerans*. While various hypotheses on potential reservoirs and vectors of *M*. *ulcerans* exist, the mode of transmission has remained unclear. Epidemiological studies have indicated that children below the age of four are less exposed to the pathogen and at lower risk of developing BU than older children. In the present study we compared the age at which children begin to develop antibody responses against *M*. *ulcerans* with the age pattern of responses to other pathogens transmitted by various mechanisms. A total of 1,352 sera from individuals living in the BU endemic Offin river valley of Ghana were included in the study. While first serological responses to the mosquito transmitted malaria parasite *Plasmodium falciparum* and to soil transmitted *Strongyloides* helminths emerged around the age of one and two years, sero-conversion for *M*. *ulcerans* and for the water transmitted trematode *Schistosoma mansoni* occurred at around four and five years, respectively. Our data suggest that exposure to *M*. *ulcerans* intensifies strongly at the age when children start to have more intense contact with the environment, outside the small movement range of young children. Further results from our serological investigations in the Offin river valley also indicate ongoing transmission of *Treponema pallidum*, the causative agent of yaws.

## Author Summary

Buruli ulcer is a debilitating skin disease caused by *Mycobacterium ulcerans*. Although the understanding of this enigmatic pathogen has improved after decades of research, the mode of transmission is yet to be fully elucidated. Recent epidemiological studies have shown an underrepresentation of Buruli ulcer cases in children below the age of four as compared to older children. In order to investigate the different exposure of children to *M*. *ulcerans* and to several other pathogens with diverse modes of transmission, we conducted a sero-epidemiological study of 1,352 residents within a five kilometer radius along the Offin River of Ghana. While our results show early exposure of children to the mosquito transmitted malaria parasite *Plasmodium falciparum* as well as to soil transmitted helminths of the genus *Strongyloides*, a later onset of immune response was observed at an age of around four and five years for *M*. *ulcerans* and the trematode *Schistosoma mansoni*, that is transmitted by contact with infested water. Similarities in age-dependent exposure of these two pathogens suggest that transmission may take place outside of the very young children’s movement range when they come into contact with the environment at the periphery of their villages.

## Introduction

Buruli ulcer (BU) is a neglected tropical skin disease presenting with a wide range of cutaneous manifestations, from non-ulcerated nodules, plaques or oedema to characteristic necrotizing ulcers [1]. While BU cases have been reported in more than 30 countries worldwide, most patients are from infection foci located in remote and rural tropical regions of West and Central Africa. BU is caused by infection with *Mycobacterium ulcerans*, a pathogen that has emerged from *M*. *marinum* by acquiring a plasmid conferring the capacity of producing the unique macrolide toxin mycolactone, accounting for much of the pathology of BU [2,3]. Until today, the mode of transmission of *M*. *ulcerans* has remained inconclusive, although proximity to aquatic habitats has long been identified as the major risk factor for contracting the disease [4]. Infection is thought to take place through either physical contact with undefined environmental reservoirs via skin abrasions or insect vectors [5–7].

It has long been recognized that in African BU endemic settings the majority of BU patients are children below 15 years of age [8]. However, a clear underrepresentation of children below the age of four becomes evident when the population age distribution is taken into account [9,10]. In line with this observation, our previous sero-epidemiological studies in Ghana and Cameroon have indicated that children below five years of age rarely develop antibody responses against the 18 kDa small heat shock protein (shsp) of *M*. *ulcerans* and thus seem to be considerably less exposed to the pathogen than older children [11]. While investigations of humoral immune responses against mycobacteria are complicated by a high degree of antigenic cross-reactivity between species, the immunodominant 18 kDa shsp overexpressed by *M*. *ulcerans* [12] represents a suitable marker for exposure to this pathogen [13]. It has no homologues in other prevalent pathogenic mycobacteria such as *M*. *bovis* and *M*. *tuberculosis* and additionally, sera from inhabitants of BU non-endemic regions generally showed no reactivity with this protein [13]. While populations living in BU non-endemic communities in proximity to the BU endemic regions seem to be similarly exposed to the 18 kDa shsp of *M*. *ulcerans* [14,15], we have observed for the three Ghanaian BU endemic river valleys Densu [15], Offin (this report) and Volta [15] an association between BU prevalence and the percentage of healthy individuals that have sero-converted.

Here we present a sero-epidemiological study carried out in the BU endemic Offin river valley of Ghana including 1,352 participants from 13 communities. The main objective was to compare the age-pattern of first humoral immune responses to the 18 kDa shsp of *M*. *ulcerans* with those to pathogens with various modes of transmission in order to contribute to our understanding of the transmission of *M*. *ulcerans*.

## Methods

### Ethics statement

Ethical clearance for the study was obtained from the institutional review board of the Noguchi Memorial Institute for Medical Research (Federal-wide Assurance number FWA00001824). Written informed consent was received from all individuals involved in the study. Parents or guardians provided written consent on behalf of their children.

### Study area

The Offin River is one of the major water bodies associated with BU in Ghana. It runs through the Ashanti and Central Regions, covering 11 health districts. In a nationwide active BU case search conducted in 1999, the Offin river valley was shown to be highly endemic for BU [16].

**Fig 1:**
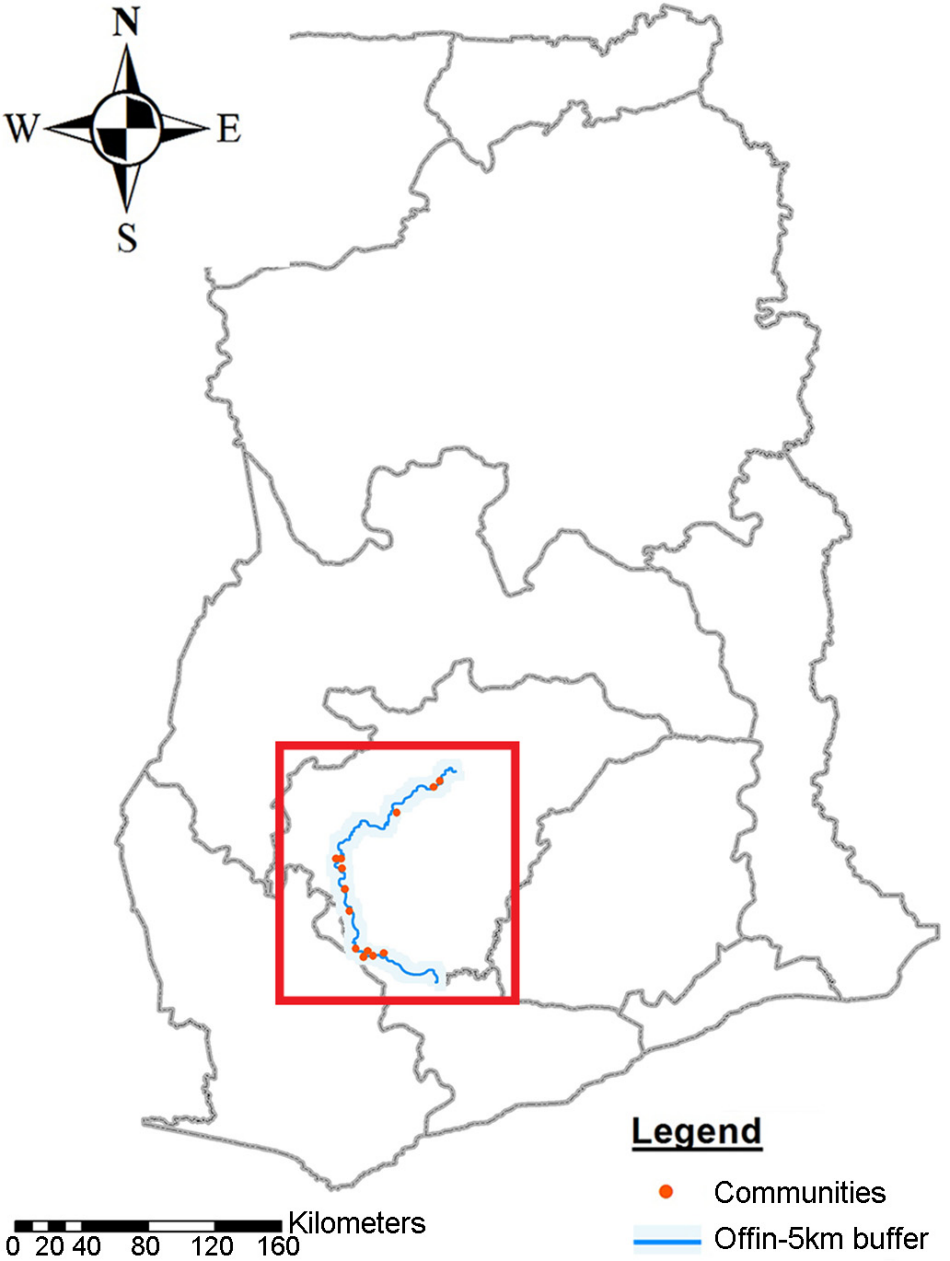
Study area. Map of the Offin river basin in Ghana with selected study communities indicated as red dots.

A total of 1,560 inhabitants of ten randomly selected communities and an additional three communities known to be BU endemic and located within a five kilometer radius along the Offin River (120 per community) (Fig 1) were randomly selected for sampling. Two milliliters of whole blood were drawn from 1,352 of 1,560 (87%) selected inhabitants in July 2013. In order to reduce dropout rates due to repetitive bleeding of participants, we randomly assigned each of the sampled individuals to one of three groups (A, B, and C) with each consisting of 450, 451, and 451 individuals to be followed up after 6 (January 2014), 12 (July 2014) or 18 (January 2015) months, respectively. Blood separation was achieved by centrifugation of the whole blood at 2’000 x g for 10 minutes. Serum was subsequently stored at -80˚C pending serological analysis. First serological analyses were performed between January and July 2014 allowing for a more thorough follow up of individual cases, such as sero-converters or sero-reverters.

### ELISA to detect anti- *M*. *ulcerans* 18 kDa shsp antibodies in serum samples

ELISA was performed as described previously [11]. Briefly, 96-well Nunc-Immuno Maxisorp plates (Thermo Scientific) were coated with 0.25 μg recombinant *M*. *ulcerans* 18 kDa shsp per well, washed with washing buffer (dH_2_O, 0.01% Tween 20) and incubated with blocking buffer (5% non-fat dry milk in PBS). Subsequently, plates were incubated with human blood serum samples (1:100 diluted). After washing, horseradish peroxidase conjugated goat anti-human IgG (γ-chain specific, SouthernBiotech) was added. Plates were washed and developed with TMB Microwell Peroxidase Substrate (KPL). The reaction was stopped using 0.16 M sulfuric acid. The absorbance was measured at 450 nm in a Tecan Sunrise microplate reader. All samples were tested in duplicates and mean values were calculated.

The cut-off value for positivity (OD_450_ = 0.25) was determined by testing serum samples with a range of ODs in ELISA by Western Blot analysis (S1 Fig). Sero-conversion/reversion was defined as a change in OD (ΔOD) between baseline and follow up samples of at least ±0.3.

### Western Blot analysis to detect anti- *M*. *ulcerans* 18 kDa shsp and anti -*P*. *falciparum* AMA-1 antibodies

Western Blot analysis was performed as described [11]. Shortly, 15 μg of recombinant *M*. *ulcerans* 18 kDa shsp or *P*. *falciparum* AMA-1 were separated on NuPAGE Novex 4–12% Bis-Tris ZOOM Gels with 1.0 mm IPG well (Invitrogen) under reducing conditions. After electrophoresis, proteins were transferred onto nitrocellulose membranes using an iBlot Gel Transfer Device (Invitrogen). Membranes were blocked with 5% non-fat dry milk in PBS containing 0.1% Tween 20 and cut into strips. Strips were then incubated with human blood sera (1:1000 dilution), washed with 0.3 M PBS containing 1% Tween 20 and thereafter incubated with horseradish peroxidase conjugated goat anti-human IgG (γ-chain specific, Southern Biotech). After a second washing step, bands were visualized by chemiluminescence using ECL Western Blotting substrate (Pierce).

### Simultaneous detection of antibodies to treponemal and non-treponemal antigens

The contagious diseases syphilis and yaws are caused by closely related *Treponema pallidum* spp. Serological diagnosis requires detection of distinct antibodies against both a treponemal antigen and a non-treponemal antigen. Non-treponemal tests become reactive during the initial stage of infection and generally revert to negative after treatment. However, treponemal antigen-based confirmation is needed, since detectable antibodies can also be due to other inflammatory conditions. Treponemal serological tests on the other hand may remain reactive for life and thus require a positive non-treponemal test result to confirm active infection [17]. Here we used the Dual Path Platform (DPP) assay manufactured by Chembio Diagnostic Systems for the simultaneous detection of antibodies to treponemal and non-treponemal antigens. This serological test, which was developed for the point-of-care diagnosis of syphilis [18], was recently also applied to and evaluated for the screening and detection of patients with yaws [19]. We tested 5 μl of the serum samples in strict accordance with the manufacturer’s instructions.

### ELISA to detect antibodies to *Schistosoma* and *Strongyloides* antigens

#### Preparation of *S*. *mansoni* antigens

Soluble egg antigen (SEA) and adult worm antigen extract (AWE) were prepared as described previously [20]. Briefly, frozen *S*. *mansoni* eggs were homogenized in PBS (pH 7.2) on ice and subsequently extracted for 3 hours at 4°C. The extract was centrifuged at 100’000 x g for 2 hours at 4°C and the supernatant was stored in aliquots at -80°C until use. Adult *S*. *mansoni* worms were homogenized in PBS (pH 7.2) containing 2 mM PMSF. The extract was centrifuged at 80’000 x g for 3 hours at 4°C and the pellet was re-suspended. After overnight incubation at 4°C the suspension was centrifuged again at 80’000 x g for 3 hours at 4°C. The supernatant was concentrated and centrifuged at 15’300 x g for five minutes at 4°C before being stored in aliquots at -80°C until use.

Both *S*. *mansoni* antigens show cross-reactivity with antibodies elicited by other *Schistosoma* ssp. (*S*. *haematobium*, *S*. *mekongi* or *S*. *japonicum*).

#### Preparation of *S*. *ratti* antigen

Mass production of antigens from *S*. *stercoralis* filariform larvae suffers the drawbacks of cost, being labor intensive and constituting a risk of infecting laboratory technicians handling the larvae. Heterologous antigen of filariform larvae of the rat parasite *S*. *ratti* provides comparable sensitivity and specificity, thereby making it a suitable alternative antigen for sero-diagnosis of human strongyloidiasis [21–23]. For this study, third-stage larvae (iL3) of *S*. *ratti* were collected as described previously [24] and frozen at -80°C. For the preparation of raw antigen, larvae were homogenized in PBS (pH 7.2) on ice. Proteins were extracted for 24 hours at 4°C on a stirrer and the suspension was centrifuged at 100’000 x g for 1 hour at 4°C. The supernatant was stored in aliquots at -80°C.

#### Detection of anti- *Schistosoma* and anti- *Strongyloides* antibodies by ELISA

Immulon 2HB plates (Thermo Labsystems) were coated with *S*. *mansoni* or *S*. *ratti* antigens in 0.05 M sodium carbonate buffer (pH 9.6) for 48 hours at 4°C. After washing with tap water containing 0.05% Tween 20, diluted sera (1:160 in PBS, pH 7.2, 0.05% Tween 20) were added to the plates and incubated for 15 minutes at 37°C. After additional washing steps, horseradish peroxidase conjugated goat anti-human IgG (KPL) was added. Plates were incubated for 15 minutes at 37°C, subsequently washed and o-Phenylendiamine dihydrochloride (OPD, Sigma) diluted in 0.6 M sodium phosphate buffer, pH 5.0 supplemented with 0.03% H_2_O_2_ was added. The reaction was stopped with 8 M H_2_SO_4_ and the absorption was read with a Multiscan FC reader (Thermo Scientific) at 492 nm. The presence of anti- *Schistosoma* antibodies was reconfirmed for the serum of a young child by immune fluorescence assay (IFA) carried out as described [20]. Serum samples positive in the *Strongyloides* ELISA were confirmed by re-testing in duplicates.

Cut-off values (Table 1) were determined previously by receiver operating characteristic (ROC) analysis of results obtained for 50 sera of healthy Swiss blood donors, 20 sera each of patients infected with *S*. *mansoni* or *S*. *stercoralis*, and 120 sera of individuals with other parasitic infections (S2 Fig). The sensitivity for the *S*. *mansoni* ELISA was 98% and 80% for AWE and SEA, respectively, while the specificity was 96% and 92% (S1 Table). SEA can exhibit cross-reactivity with *Trichinella* and to a lesser extent with *Filaria*. The sensitivity of the *S*. *ratti* ELISA was 95%, while the specificity was 84% due to potential cross-reactivity with *Filaria* and *Echinococcus* ssp (S1 Table).

**Table 1:** Cut-off ODs for the *S*. *mansoni* and *S*. *ratti* ELISA.

Pathogen	Antigen	ELISA cut-off (OD)		
		Negative	Equivocal	Positive
*S*. *mansoni*	SEA^a^	< 0.3	0.3–0.59	≥ 0.6
	AWE^b^	< 0.15	0.15–0.29	≥ 0.3
*S*. *ratti*	raw antigen	< 0.50	0.50–0.69	≥ 0.70

^a^Soluble egg antigen

^b^Adult worm antigen extract

### Data analysis

ELISA results were analyzed using GraphPad Prism version 6.0 (GraphPad Software, San Diego California USA). The distribution of antibody titers is presented as box plots. These comprise a line for the median, edges for the 25th and 75th percentiles and traditional Tukey whiskers showing 1.5 times the interquartile distance. Dots on the graph represent individual points that lie outside that range. The overall difference and variation between samples tested in duplicates was estimated using the Bland-Altman method [25].

## Results

### Age distribution of *M*. *ulcerans* 18 kDa shsp specific serum IgG responses

In order to assess exposure of the population living in a five kilometer radius along the Offin River to *M*. *ulcerans*, serum samples taken from 1,352 study participants of 13 communities across different age groups (Table 2) were tested in duplicates for the presence of anti- *M*. *ulcerans* 18 kDa shsp IgG by ELISA. Between the two duplicate test results, a negligible overall bias with a mean difference (OD1-OD2) of 0.004 was estimated. The variation in individual differences was very small, with 95% limits of agreement from −0.095 to 0.103. At baseline, 18% of the serum samples contained IgG titers against the *M*. *ulcerans* protein. In all, 3% (46/1,352) of the participants were BU cases with healed or active lesions and 13% (179/1,352) were household contacts of BU patients. Amongst the BU cases, 24% (11/46) had antibodies against the *M*. *ulcerans* 18 kDa shsp, while 15% (26/179) of the household contacts contained anti- *M*. *ulcerans* 18 kDa shsp titers in their sera.

**Table 2:** Population, study participants and serum positivity for the different antigens tested.

Variables		Age in years															
		1^a^	2	3	4	5	6	7	8	9	10	11–20	21–30	31–40	41–50	51–60	>60
n = Total population (%)		544 (2.7)	694 (3.4)	645 (3.2)	661 (3.3)	570 (2.8)	534 (2.7)	546 (2.7)	570 (2.8)	487 (2.4)	614 (3.1)	4,598 (22.8)	3,827 (19)	2,335 (11.6)	1,507 (7.5)	1,019 (5.1)	976 (4.8)
n = Participants (% of population)		3 (0.6)	16 (2.3)	47 (7.3)	52 (7.9)	52 (9.1)	35 (6.6)	45 (8.2)	55 (9.6)	41 (8.4)	56 (9.1)	306 (6.7)	183 (4.8)	170 (7.3)	110 (7.3)	96 (9.4)	85 (8.7)
n = anti- *M*. *ulcerans* 18 kDa shsp pos. (% of participants)		0 (0)	0 (0)	1 (2)	3 (6)	2 (4)	2 (6)	3 (7)	8 (15)	3 (7)	9 (16)	49 (16)	34 (19)	43 (25)	30 (27)	25 (26)	20 (24)
n = anti- *P*. *falciparum* AMA-1 pos. (%)		1/3 (33)	7/15 (47)	26/37 (70)	—-	—-	—-	—-	—-	—-	—-	—-	—-	—-	—-	—-	—-
n = anti- *T*. *pallidum* pos. (%)	Both antigens	0 (0)	0 (0)	1 (2)	0 (0)	0 (0)	0 (0)	1 (2)	0 (0)	1 (2)	1 (2)	5/142 (4)	1/13 (8)	1/14 (7)	1/15 (7)	1/14 (7)	6/19 (32)
	Treponemal antigen only	0 (0)	0 (0)	0 (0)	0 (0)	3 (6)	0 (0)	1 (2)	1 (2)	1 (2)	3 (5)	4/142 (3)	2/13 (15)	0 (0)	2/15 (13)	0/14 (0)	5/19 (26)
n = anti- *Strongyloides* spp. pos. (%)		0 (0)	1 (6)	1 (2)	1 (2)	1 (2)	1 (3)	0 (0)	1 (2)	2 (5)	4 (7)	15/142 (11)	0/13 (0)	1/14 (7)	3/15 (20)	1/14 (7)	1/19 (5)
n = anti- *S*. *mansoni* pos. (%)	SEA^b^	0 (0)	0 (0)	0 (0)	0 (0)	1 (2)	1 (3)	1 (2)	3 (5)	0 (0)	1 (2)	16/142 (11)	2/13 (15)	1/14 (7)	2/15 (13)	0/14 (0)	1/19 (5)
	AWE^c^	0 (0)	0 (0)	1 (2)	0 (0)	0 (0)	0 (0)	1 (2)	2 (4)	0 (0)	2 (4)	15/142 (11)	2/13 (15)	4/14 (29)	3/15 (20)	0/14 (0)	2/19 (11)

Total numbers and percentages of the study population, study participants and the proportions of serum samples tested positive in the different serological tests are shown.

^a^no child below one year participated in the study

^b^soluble egg antigen

^c^adult worm antigen extract

**Fig 2:**
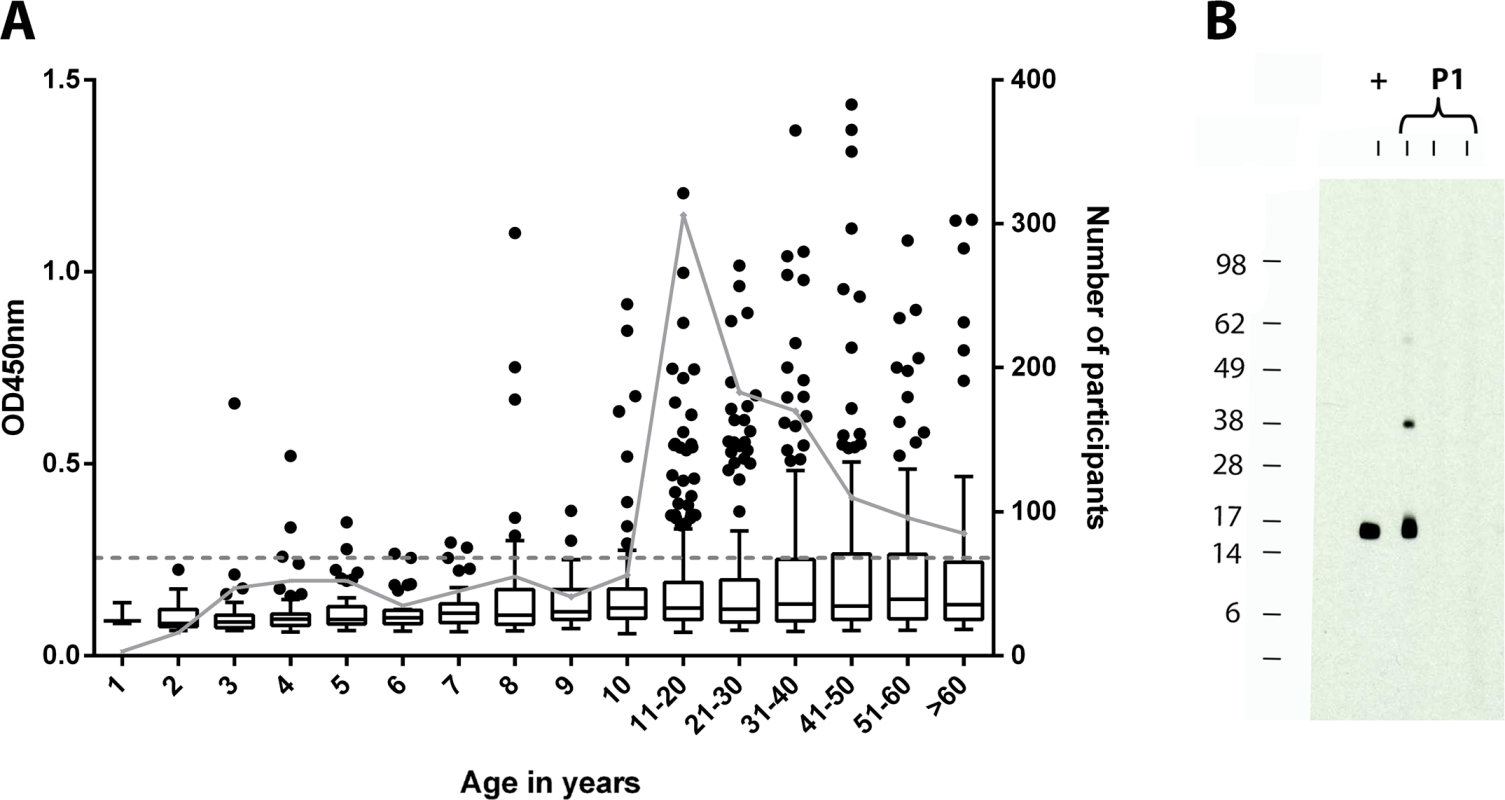
Age distribution of anti- *M*. *ulcerans* 18 kDa shsp serum IgG titers. (A) Boxplots of OD values of 1:100 diluted serum samples tested in the anti- *M*. *ulcerans* 18 kDa shsp IgG specific ELISA by age group. The background cut-off value (OD<0.25) is indicated as a dotted line. The number of study participants for each age group (right y-axis) is visualized by grey dots connected by a grey line. (B) Sera obtained from a child participant (P1) at three different time points were tested for the presence of anti- *M*. *ulcerans* 18 kDa shsp serum IgG by Western Blot analysis. While the serum of the child tested positive at baseline, sero-reversion was observed after it had moved to a BU non-endemic community. A positive control serum (+) was included. The molecular weight standard (in kDa) is shown on the left of the blot.

The age distribution of the mean of duplicate anti- *M*. *ulcerans* 18 kDa shsp IgG titers for the study participants is shown in Fig 2A and Table 2. While in all age groups above nine years individuals with high IgG titers were detected, only three sera from children below the age of five years contained 18 kDa shsp specific antibodies and also most of the sera from children between five and seven years tested negative. The only child below four years of age with positive test result was a three year old resident of Mfantseman, a BU endemic community. We carefully followed up this particularly interesting individual case at two occasions. Before May 2013, he lived for his entire life in a BU non-endemic community (Wanpiem) not included in this study, but was transferred thereafter to the BU endemic community Mfantseman for schooling and was enrolled there in this study. Shortly after sampling his blood in July 2013, he was relocated to Wanpiem by his guardian. Scheduled for sampling after 12 months (July 2014), we followed him up at Wanpiem and observed that he had sero-reverted. His serum still tested negative for anti- *M*. *ulcerans* 18 kDa shsp IgG after 18 months of follow up (January 2015). Thus, his total length of stay in the BU endemic Mfantseman community was three months (May-July 2013); coinciding with the only sampling point at which we observed a high anti- *M*. *ulcerans* 18 kDa shsp IgG titer in his serum. Western blot results for the three time points reconfirmed the ELISA data (Fig 2B). Testing of sera from all other study participants younger than five years by Western Blot analysis revealed no specific bands (representing IgG titers) against the 18 kDa shsp for sera from children below four years of age (S3 Fig), while positive sera were detected in all age groups ≥4 years (S3 and S4 Figs), indicating that sero-conversion may start in some children around the age of four years.

### Stability of anti- *M*. *ulcerans* 18 kDa shsp IgG titers

**Fig 3:**
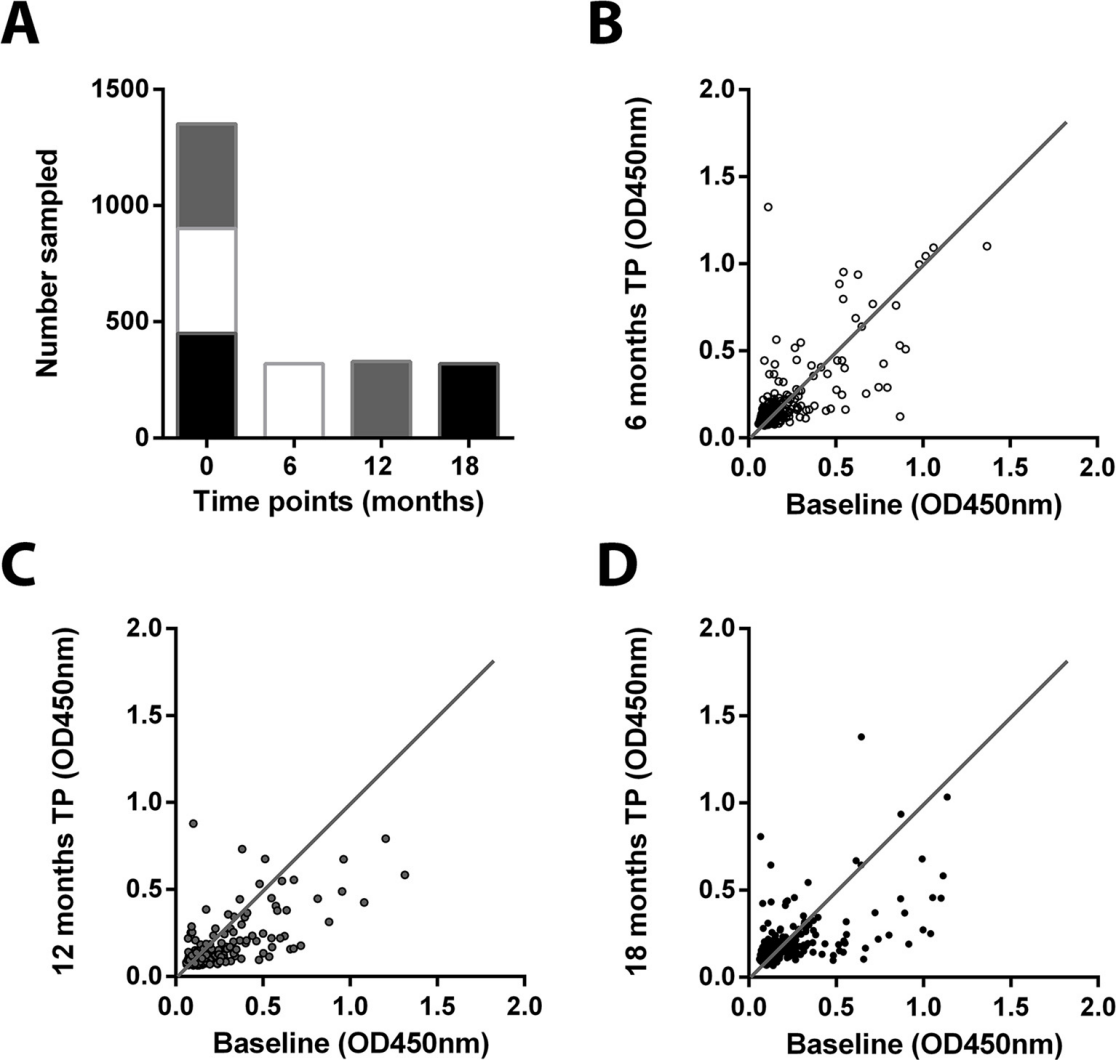
Design and results of a longitudinal sero-epidemiological study. (A) Blood samples were taken in July 2013 from 1,352 inhabitants of the communities selected. All sampled individuals were followed up after either 6 (January 2014), 12 (August 2014) or 18 (January 2015) months. IgG titers against the *M*. *ulcerans* 18 kDa shsp were determined in serum samples collected at baseline and compared to titers in samples collected at different time points (TP) after 6 (B), 12 (C) or 18 (D) months. A straight line was drawn to visualize deviations between time points analyzed.

Follow up samples were taken from 319 of 450 (71%), 329 of 451 (73%), and 356 of 451 (79%) participants assigned to groups A, B and C after 6, 12, or 18 months, respectively (Fig 3A). Testing of the serum samples for the presence of anti- *M*. *ulcerans* 18 kDa shsp IgG revealed that based on the defined value for the difference between paired samplings (ΔOD = 0.3), antibody titers remained stable for 98%, 97%, and 92% of the study participants, respectively. After 6, 12, and 18 months 1%, 0.3%, and 6% of the sero-positive individuals had sero-reverted, while 1%, 3% and 1.4% of the sero-negative participants had sero-converted (Fig 3B–3D).

### Age distribution of IgG titers against antigens of other pathogens with known mechanism of transmission

#### Plasmodium falciparum

**Fig 4:**
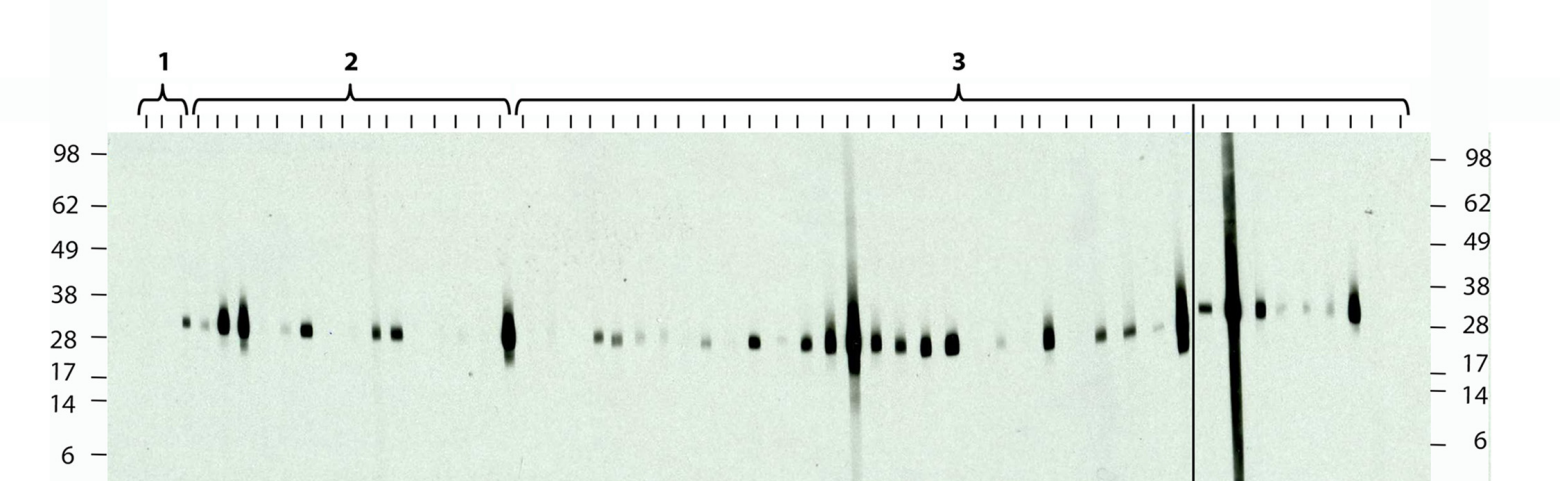
Western blot analysis of anti- *P*. *falciparum* AMA-1 serum IgG responses in young children aged one (1), two (2) and three (3) years. A number of sera from children aged one and two years already contained serum IgG against recombinant *P*. *falciparum* AMA-1 protein, as determined by Western Blot analysis. The molecular weight standard (in kDa) is shown next to the two blots that are separated by a vertical black line.

Serum samples collected from children below the age of four years were tested for the presence of IgG titers against the AMA-1 protein of the mosquito transmitted malaria parasite *P*. *falciparum*. In contrast to the late onset of the serological response in children for the *M*. *ulcerans* 18 kDa shsp, Western Blot analysis for AMA-1 showed that a number of sera from one (1/3) and two (7/15) year old infants and almost all sera from three year old children (26/37) contained antibodies against this microneme protein of sporozoites and merozoites [26] (Fig 4, Table 2).

#### Treponema pallidum

Serum samples from all children below the age of 11 years (n = 402) and representatives of older age groups (n = 217) were analyzed for the presence of antibodies against non-treponemal and treponemal antigens using the DPP Screen and Confirm assay (Chembio Diagnostic Systems). Antibodies against both treponemal and non-treponemal antigens were detected in sera from only a limited number of children below the age of 11 years (4/402 children, 1%). Sera from nine children below the age of 11 years (9/402, 2.2%) tested positive for the treponemal antigen only. A higher percentage of sera from older children and adults tested positive for both antigens (15/217; 6.9%) as well as for the treponemal antigen alone (13/217; 6%) (Table 2).

#### Strongyloides spp

**Fig 5:**
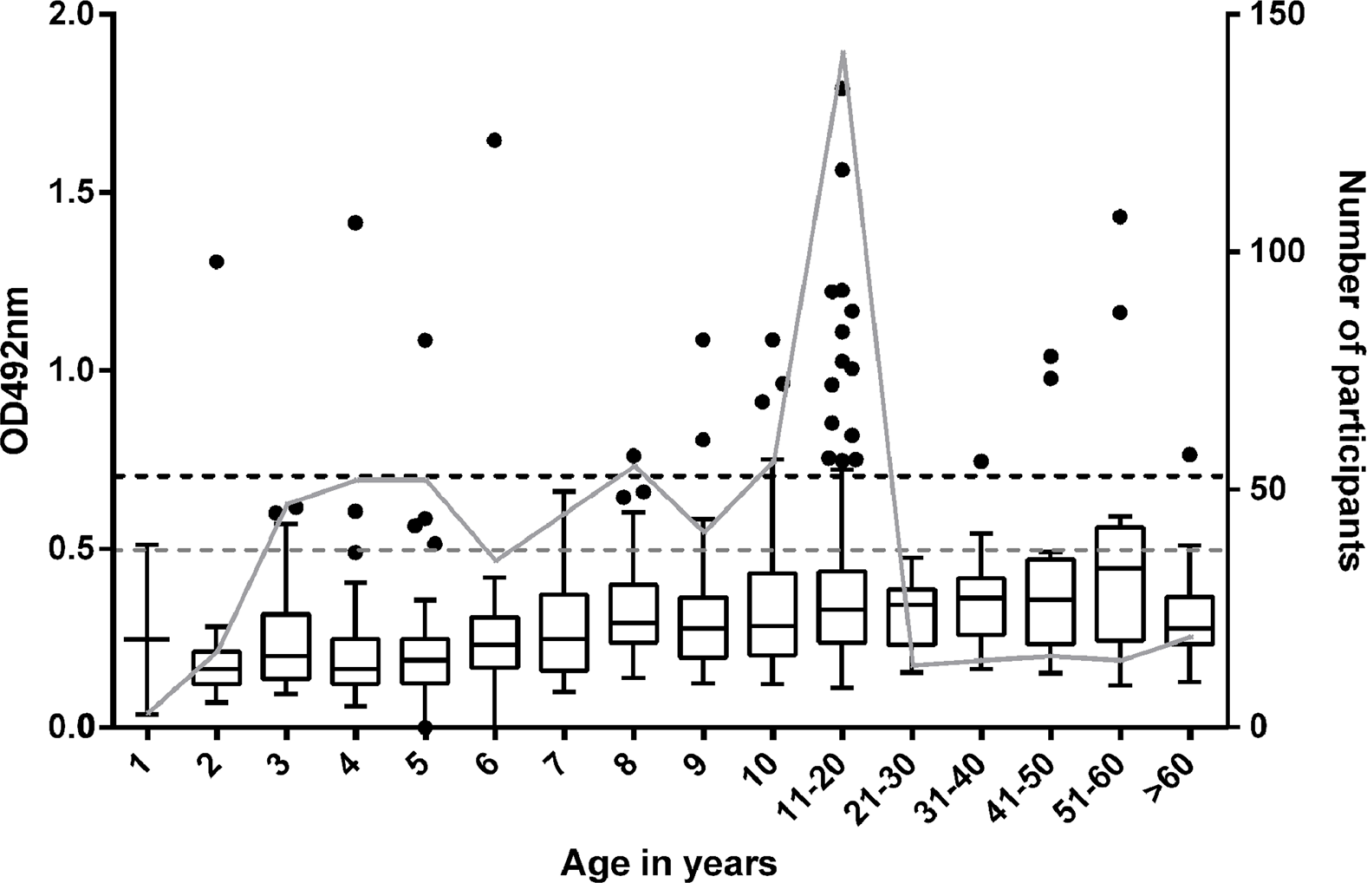
Age distribution of anti- *Strongyloides* serum IgG titers. All sera from children below the age of 11 years as well as a subset of sera from older age groups were tested for the presence of anti- *Strongyloides* antigen IgG by ELISA. The grey and the black dotted line indicate the cut-off OD values for equivocal and positive test results, respectively. The number of study participants for each age group (right y-axis) is visualized by grey dots connected by a grey line.

Sera from all children below 11 years of age and from the same representatives of older age groups as tested for antibodies against *T*. *pallidum* antigens were also analyzed for IgG titers against raw antigens of *S*. *ratti* by ELISA as a marker for exposure to *S*. *stercoralis*, since *S*. *ratti* antigen is cross-reactive with antibodies elicited by infection with this soil-transmitted helminth. The overall sero-positivity was low with 41/619 (6.6%) sera yielding equivocal test result and 33/619 (5.3%) testing positive. However, 3/170 (1.8%) children aged five years and below already tested positive (Fig 5, Table 2), demonstrating early exposure to infested soil.

#### Schistosoma mansoni

**Fig 6:**
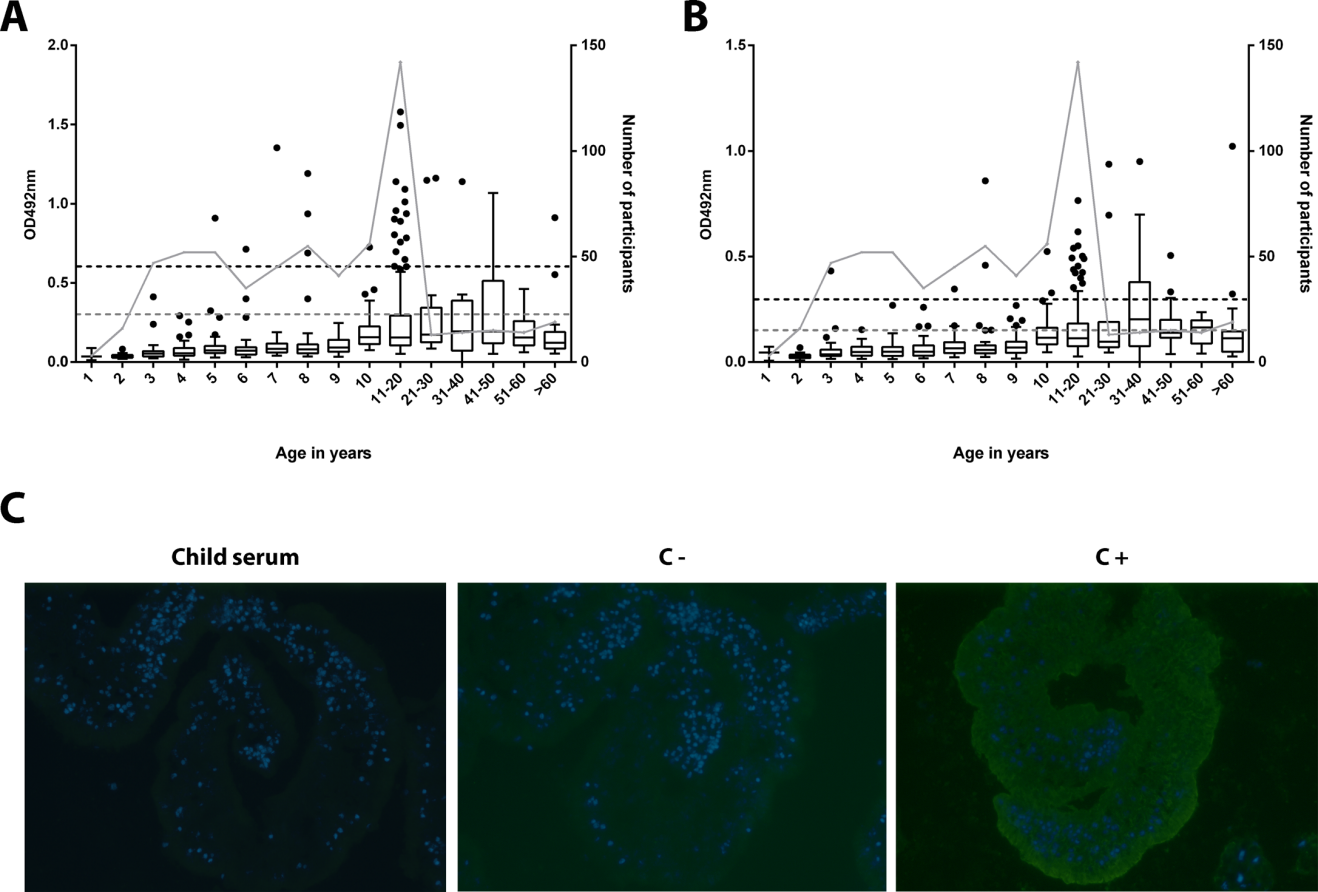
Age distribution of anti- *S*. *mansoni* SEA and AWE serum IgG titers. All sera from children below the age of 11 years and a subset of individuals of older age groups were tested for the presence of IgG against *S*. *mansoni* SEA (A) and AWE (B) by ELISA. The grey and the black dotted line indicate the cut-off OD values for equivocal and positive test results, respectively. The number of study participants for each age group (right y-axis) is visualized by grey dots connected by a grey line. The serum of the only young child testing positive in the AWE ELISA was reconfirmed to be positive by IFA. The serum of this child as well as a negative control serum labelled C- and a positive control serum labelled C+ is shown (C).

Serum samples from all children <11 years and from the same representatives of older age groups as tested for antibodies against the other antigens were also analyzed for the presence of IgG against SEA and AWE of *S*. *mansoni*. Of the 619 sera, 29 (4.7%) and 32 (5.2%) tested positive in the SEA and AWE ELISA, respectively. Most of the responders (n = 18; 2.9%) tested positive in both assays. While none of the children below the age of five tested positive against *S*. *mansoni* SEA, seven children aged five to ten had sero-converted and 16/142 (11.3%) individuals aged between 11 and 20 had developed high anti-SEA antibody titers (Fig 6A, Table 2). A similar pattern was observed for the AWE with only one child aged below seven testing positive (Fig 6B, Table 2). The serum of this child was reconfirmed as being weakly positive by IFA (Fig 6C).

## Discussion

Since only a minority of individuals exposed to *M*. *ulcerans* develops clinical BU, sero-epidemiological studies represent a valuable tool to assess the exposure of populations in BU endemic areas to the pathogen. In line with data obtained from previous sero-epidemiological investigations in BU endemic areas located in the Densu river basin of Ghana and in the Mapé river basin of Cameroon [11,15], we reconfirmed here for the population of the BU endemic Offin river valley that young children below four years of age are considerably less exposed to *M*. *ulcerans* than older children. In contrast, our serological data showed, as expected, that exposure to the mosquito transmitted malaria parasite *P*. *falciparum* and to soil-transmitted helminths of the genus *Strongyloides* takes place already in very young children, as indicated by an early development of humoral immune responses against these pathogens in some of the infants. The delay in exposure of *M*. *ulcerans* and the relatively abrupt onset are in stark contrast to the age-patterns for *Plasmodium* and *Strongyloides*. Contact with larvae-infested soil through faecal contamination is likely to be responsible for the observed early development of anti- *Strongyloides* serum IgG responses. Our results suggest that contact with *M*. *ulcerans* occurs outside the small movement range of infants, providing indirect evidence against mechanisms of transmission involving vectors or reservoirs present in the vicinity of the children’s homes. However, our data do not exclude an involvement of insect vectors commonly found at the periphery of villages close to water contact sites. We recognize that the age distribution of anti- *P*. *falciparum* serum antibodies depends not only on the mode of transmission, but also on the transmission intensity [27,28]. While there is some evidence that mosquitos may be involved in transmission of *M*. *ulcerans* in south-eastern Australia [29,30], it is overall unlikely that they play a major role as vectors in African BU endemic settings. This assumption is also supported by previous molecular epidemiological studies showing that transmission of newly emerging genetic variants of *M*. *ulcerans* is geographically highly clustered [31,32].

In Ghana schistosomiasis is mainly caused by *S*. *haematobium* and *S*. *mansoni* [33]. The egg forms of the parasite are shed into the environment via urine or faeces of an infected person. Through a complex life cycle involving an intermediate snail host, a healthy individual can be infected by coming into contact with water sources infested with invasive larvae [34]. Therefore, the risk of infection is related to water contact patterns. Peak prevalence is usually observed in school-aged children, but may be shifted to adulthood depending on the degree of endemicity in a setting [35]. In the present study we observed in the Offin river basin the typical age distribution of *Schistosoma* exposure with children below the age of eight being significantly less affected than older children and young adults. This pattern was similar to the age distribution of exposure to *M*. *ulcerans* in the same population. While the mechanisms of infection by schistosomes and *M*. *ulcerans* are likely to be different, similarities in the age-dependent patterns of exposure may be related to changes in water contact patterns. It remains to be elucidated whether infection with *M*. *ulcerans* from an environmental reservoir takes place through skin lesions or via invertebrate vectors, such as aquatic insects. In addition, our data indicate that anti- *M*. *ulcerans* 18 kDa shsp IgG titers are relatively stable with only 2%, 3% and 8% of individuals followed up after 6, 12 and 18 months, respectively, having sero-converted or sero-reverted. A limitation of this longitudinal study was that study participants were followed up only once after 6, 12 or 18 months. This study design was chosen to reduce dropout rates of study participants due to repeated blood drawings. Follow up of all study participants at three time points would have strengthened conclusions on the stability of antibody titers and in combination with environmental studies might have shed further light onto contact patterns of individuals with *M*. *ulcerans*.

As part of our study we also analyzed serological responses of children below the age of 11 years to treponemal and non-treponemal antigens in order to assess exposure to *T*. *pallidum* subspecies *pertenue*, the causative agent of yaws, which is transmitted by direct contact with the fluid from the lesion of an infected person. In contrast to syphilis, caused by the closely related *T*. *pallidum* subspecies *pallidum*, yaws mainly affects children living in poor rural areas of tropical countries [36]. Traditionally, the recommended algorithm for the serological diagnosis of treponemal diseases includes a non-treponemal test for screening, and a treponemal test for confirmation. Non-treponemal tests detect antibodies to non-treponemal antigens such as cardiolipin and lecithin released from damaged host cells or lipoprotein-like material released from the treponemes. Due to the occurrence of false-positive reactions, treponemal test results based on *T*. *pallidum* antigen are required for a reconfirmation of non-treponemal tests [37].

While eradication campaigns in the 1950s and 1960s by mass treatment of affected communities led to a drastic reduction of worldwide cases, yaws has lately re-emerged in Africa, Asia and the western Pacific [38] and Ghana was recently reported to be among the three most endemic countries for yaws [39]. Official case notification rates were 32 and 383 per 100,000 population in 2010 for the Ashanti and Central regions, respectively [40], but underreporting is suspected.

In this study antibodies to non-treponemal and treponemal antigens were detected in four of 402 (1%) children below the age of 11, indicating active yaws transmission in the affected communities. Antibodies to the treponemal antigen only were found in 2.2% of the children. Since yaws and syphilis are serologically indistinguishable, the interpretation of test results in adolescents and adults would require careful clinical assessment. While a recently published study in the Northern Region of Ghana has not found evidence of active yaws despite of continued case reporting [41], our data demonstrate evidence of ongoing yaws transmission in communities of the Offin river valley. There is an urgent need for more comprehensive data on the prevalence of yaws in Ghana to better implement mass drug administration programs.

Comparative genome analyses, environmental studies, as well as serological and epidemiological studies of BU affected populations have in the last decades gradually broadened our knowledge of environmental reservoirs and probable infection routes of *M*. *ulcerans*. Future longitudinal sero-epidemiological and environmental studies over longer time periods combined with the monitoring of environmental contact patterns may be required to unravel mysteries of *M*. *ulcerans* transmission.

## Supporting Information

S1 Fig Western Blot analysis of serum samples with a range of ODs in ELISA. (TIF)

S2 Fig Detection of IgG against antigens of *S*. *ratti* iL3 (A), *S*. *mansoni* AWE (B) and *S*. *mansoni* SEA (C) in serum of parasitologically confirmed strongyloidiasis patients (A, Strongyloides), serum of parasitologically confirmed schistosomiasis patients (B and C, Schistosoma), negative control individuals (Blood donors) and patients with other parasitic infections (Other parasites). Each symbol shows a single serum sample. The corresponding cut-off is indicated in each graph by a horizontal line. The cut-off for the *Strongyloides* ELISA is 0.7, for *Schistosoma* AWE 0.30 and for *Schistosoma* SEA 0.60. n = number of samples.
(TIF)

S3 Fig Western Blot analysis of sera from all children <5 years for anti-18 kDa shsp IgG responses. (TIF)

S4 Fig Western Blot analysis of sera from a number of children >4 years for anti- *M*. *ulcerans* 18 kDa shsp IgG responses. (TIF)

S1 Table Sensitivity and Specificity of ELISAs. The sensitivity and specificity with 95% CI (confidence interval) and the AUC (area under the ROC curve) values for the *S*. *mansoni* and *S*. *ratti* ELISAs are shown.
(DOCX)
