# Supplementary figures and images for: A Sero-epidemiological Approach to Explore Transmission of Mycobacterium ulcerans
Source: PLoS Negl Trop Dis. 2016 Jan 25;10(1):e0004387. doi: 10.1371/journal.pntd.0004387 (PMC4726553; doi:10.1371/journal.pntd.0004387)

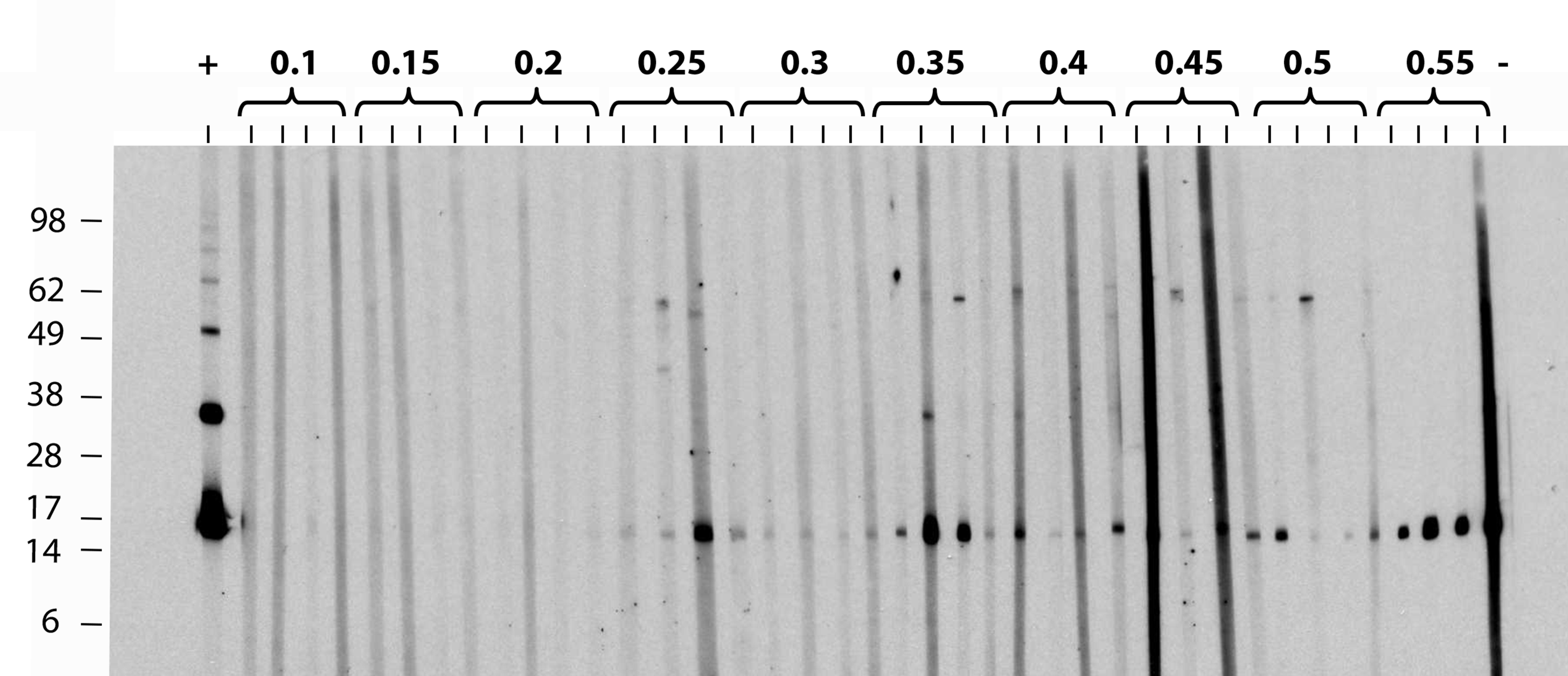

Supplement: S1 Fig — (TIF) [file pntd.0004387.s001.tif]

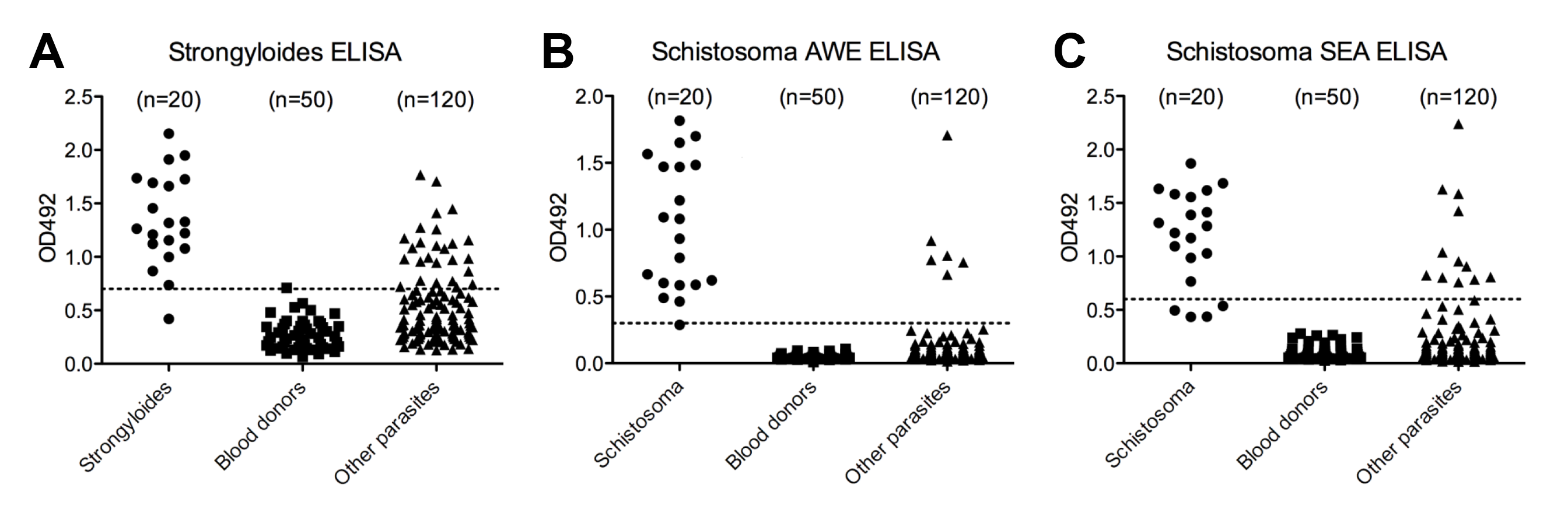

Supplement: S2 Fig — Each symbol shows a single serum sample. The corresponding cut-off is indicated in each graph by a horizontal line. The cut-off for the Strongyloides ELISA is 0.7, for Schistosoma AWE 0.30 and for Schistosoma SEA 0.60. n = number of samples. (TIF) [file pntd.0004387.s002.tif]

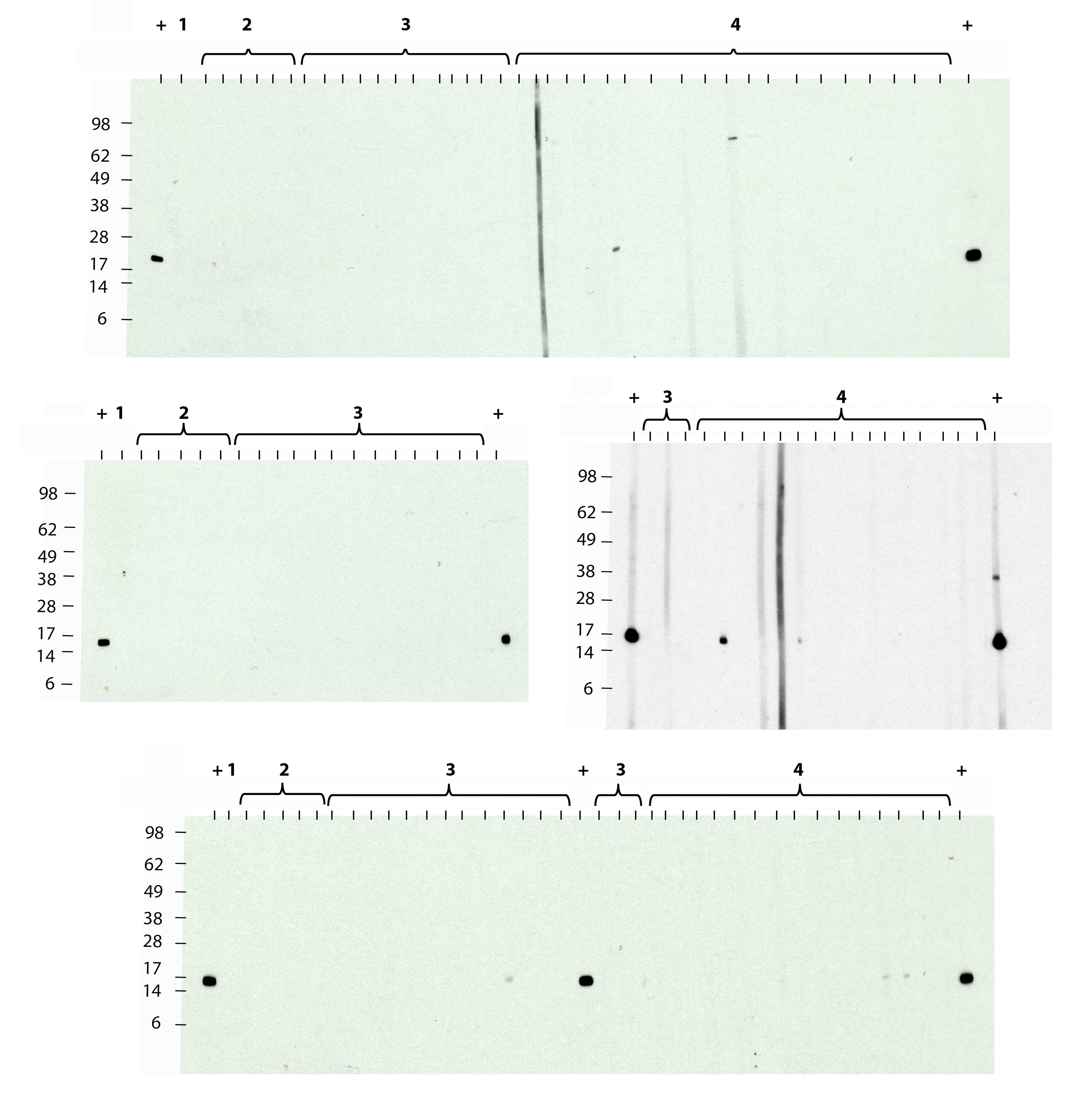

Supplement: S3 Fig — (TIF) [file pntd.0004387.s003.tif]

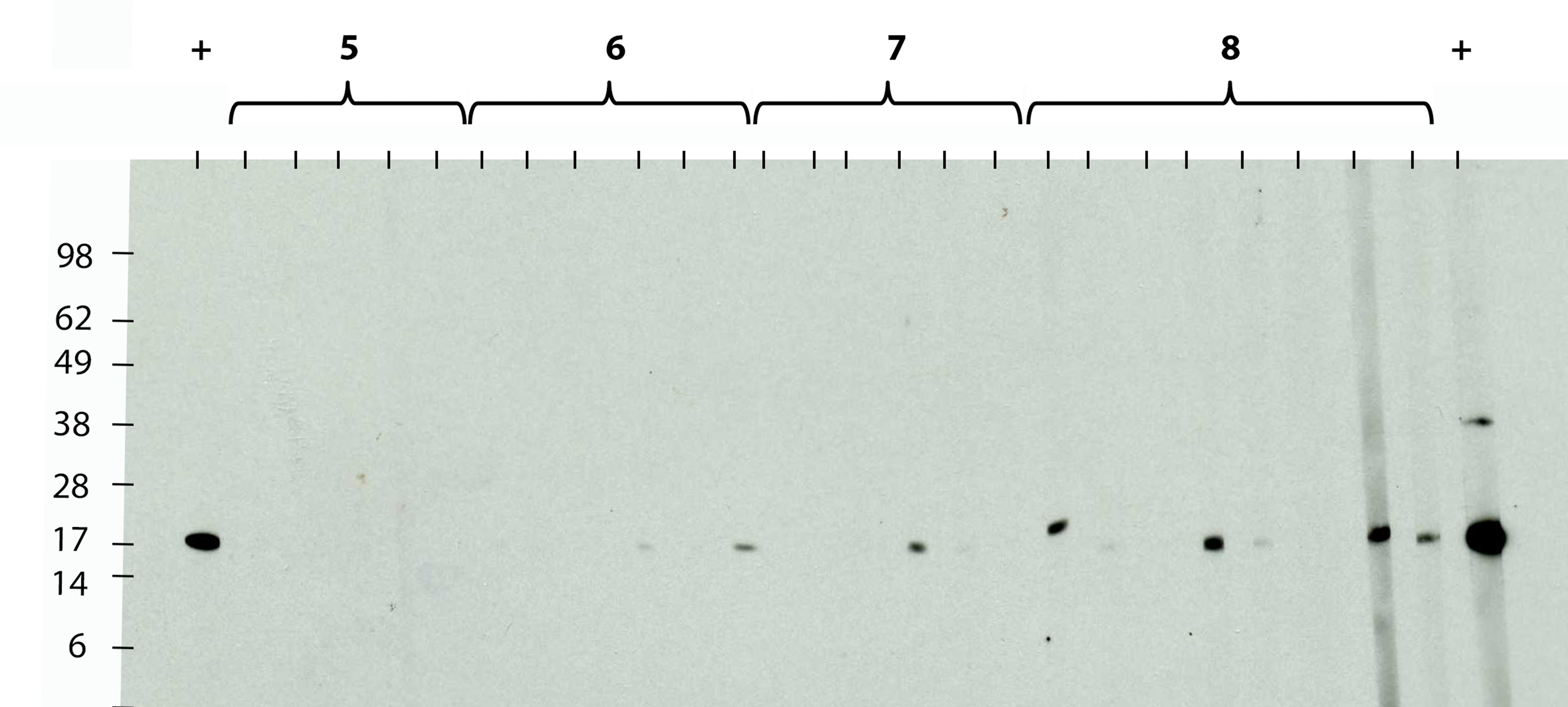

Supplement: S4 Fig — (TIF) [file pntd.0004387.s004.tif]
