# Supplementary material for: A Sero-epidemiological Approach to Explore Transmission of Mycobacterium ulcerans
Source: PLoS Negl Trop Dis. 2016 Jan 25;10(1):e0004387. doi: 10.1371/journal.pntd.0004387 (PMC4726553; doi:10.1371/journal.pntd.0004387)
Supplement: S1 Table — The sensitivity and specificity with 95% CI (confidence interval) and the AUC (area under the ROC curve) values for the S. mansoni and S. ratti ELISAs are shown. (DOCX) [file pntd.0004387.s005.docx]

**Table S1: Sensitivity and Specificity of ELISAs**

| ELISA | Sensitivity (%) (95% CI) | Specificity (%) (95% CI) | ROC AUC |
| --- | --- | --- | --- |
| Strongyloides | 95.0 (75.1-99.9) | 84.3 (78.4-89.1) | 0.95 |
| Schistosoma AWE | 97.5 (79.2-99.9) | 95.9 (92.2-98.2) | 0.98 |
| Schistosoma SEA | 80.0 (56.3-94.3) | 92.4 (87.8-95.7) | 0.96 |
